# The Effects Analysis of Contact Stiffness of Double-Row Tapered Roller Bearing under Composite Loads

**DOI:** 10.3390/s23104967

**Published:** 2023-05-22

**Authors:** Fanyu Zhang, Hangyuan Lv, Qingkai Han, Mingqi Li

**Affiliations:** 1 Foshan Graduate School of Innovation, Northeastern University, Foshan 528311, China; 2210128@stu.neu.edu.cn (F.Z.);; 2 School of Mechanical Engineering and Automation, Northeastern University, Shenyang 110819, China; 3 Key Laboratory of Vibration and Control of Aero Propulsion Systems Ministry of Education of China, Northeastern University, Shenyang 110819, China

**Keywords:** double-row tapered roller bearing, contact stiffness, composite load, pretightening force

## Abstract

Double-row tapered roller bearings have been widely used in various equipment recently due to their compact structure and ability to withstand large loads. The dynamic stiffness is composed of contact stiffness, oil film stiffness and support stiffness, and the contact stiffness has the most significant influence on the dynamic performance of the bearing. There are few studies on the contact stiffness of double-row tapered roller bearings. Firstly, the contact mechanics calculation model of double-row tapered roller bearing under composite loads has been established. On this basis, the influence of load distribution of double-row tapered roller bearing is analyzed, and the calculation model of contact stiffness of double-row tapered roller bearing is obtained according to the relationship between overall stiffness and local stiffness of bearing. Based on the established stiffness model, the influence of different working conditions on the contact stiffness of the bearing is simulated and analyzed, and the effects of radial load, axial load, bending moment load, speed, preload, and deflection angle on the contact stiffness of double row tapered roller bearings have been revealed. Finally, by comparing the results with Adams simulation results, the error is within 8%, which verifies the validity and accuracy of the proposed model and method. The research content of this paper provides theoretical support for the design of double-row tapered roller bearings and the identification of bearing performance parameters under complex loads.

## 1. Introduction

Double-row tapered roller bearing is a kind of double-row roller bearing with a tapered raceway. Because of its compact structure and good bearing performance, it is widely used in railway freight cars [1,2] and high-speed railway passenger cars [3,4]. The structure and load-bearing characteristics of double-row tapered roller bearings make the internal mechanism very complex. In addition, the working environment is harsh, the load-bearing is complicated, and the installation space is narrow, so it is very difficult to analyze its dynamic performance. The stiffness reflects the ability of the instrument parts to resist deformation during operation, and the dynamic analysis of stiffness will provide theoretical support for the design and application of instrument parts [5,6]. Stiffness of bearings is an important parameter that determines the dynamic characteristics of the bearing and the dynamic characteristics of the rotor system [7,8,9,10]. Therefore, it is of great significance to study the stiffness characteristics of double-row tapered roller bearings to ensure the safe and stable operation of railway freight cars and high-speed railways.

In the study of rotor-bearing systems, the bearing is often only equivalent to a constant stiffness spring, ignoring the nonlinear variation of bearing stiffness with load and speed [11,12]. With the increase in system speed and load, the nonlinear characteristics of stiffness are more apparent, and the research on bearing stiffness begins to increase. Aiming at the stiffness problem of ball bearings, Hemot [13], Sheng [14] and Guo [8] established the contact stiffness calculation model of ball bearings. Cao [15] studied the influence of ball number, preload, radial load, and rotational speed on the time-varying stiffness of ball bearings. Geng [16] established a five-degree-of-freedom quasi-static model of double-row self-aligning ball bearings and studied the influence of angular misalignment on bearing stiffness. Zhang [17] proposed a quasi-static model of angular contact ball bearings considering the centrifugal force and gyroscopic effect and studied the change in stiffness under different preload forms. The results show that the stiffness of the bearing under constant pressure preload decreases significantly with the increase of rotational speed, while the stiffness under positioning preload does not decline significantly. Xu [18] established a comprehensive multi-degree-of-freedom mathematical model for double angular contact ball bearings and studied the influence of single axial load, single radial load, combined load, and misalignment angle on stiffness characteristics. The results show that the misalignment effect depends on the radial load and the misalignment effect significantly affects the stiffness characteristics of the bearing. In [19], the influence of rotational speed on the stiffness of ball bearing under three typical load conditions was studied comprehensively and systematically, and the effects of bearing inner clearance, ball diameter, inner and outer raceway curvature coefficient on the stiffness variation law and rotational speed of ball bearing under three typical load conditions were given. Aiming at the stiffness problem of cylindrical roller bearings, Natalia [20], Xu [21], and Xu [22] established the contact stiffness calculation models of cylindrical roller bearings. Based on modeling, Xu [22] discussed the influence of different defect width angles on time-varying stiffness. Meng [23] analyzed the influence of radial clearance on the stiffness of high-speed cylindrical roller bearings. Huang [24] established a bearing stiffness model based on quasi-static analysis. In the model, the centrifugal effect and elastohydrodynamic lubrication effect of rollers were considered to establish high-speed cylindrical roller bearings. Zheng [25] proposed a new method to analyze the non-uniform load distribution and stiffness of rollers. The contact angle curve of each raceway was studied in depth, and the contact angle, non-uniform load distribution, energy loss, and stiffness with external load and preload were discussed. Aiming at the stiffness problem of tapered roller bearings, Lim [26] gave the calculation equations of radial contact stiffness and axial contact stiffness of single-row tapered bearings. Zantopulos [27] derived the radial and axial stiffness of single-row tapered roller bearings under constant load based on Sjovall’s radial and axial elastic deformation equations. Based on the quasi-static analysis, Tong [28,29] gave the stiffness calculation model of single row tapered bearing, and studied the stiffness characteristics of the bearing. The results show that the stiffness of the bearing is obviously affected by the rotational speed, and increasing the axial preload can significantly improve the stiffness of the tapered roller bearing. It can be seen that the current research on bearing stiffness mainly focuses on ball bearings and cylindrical roller bearings, with a few studies on the stiffness of single-row tapered bearings; the research on the stiffness characteristics of double-row tapered bearings is rare, and the influence of stiffness characteristics is not apparent. Therefore, it is very important to study the stiffness of double-row tapered roller bearings for bearing design and bearing fault diagnosis [30,31].

In this paper, the contact mechanics calculation model of double-row tapered roller bearing under composite load and the calculation method of local contact stiffness of tapered bearing are offered. On this basis, the calculation model of the overall stiffness of the bearing is obtained according to the relationship between the overall stiffness and local stiffness of the bearing. Then, according to the established model, the influence of working parameters (including axial load, radial load, bending moment load, rotational speed, preload, deflection angle, etc.) on the radial and axial contact stiffness of double-row tapered roller bearings is analyzed. The applicability and reliability of the model are verified via finite element simulation.

## 2. Calculation Method for Contact Stiffness of Double-Row Tapered Roller Bearings

### 2.1. Force Analysis of Double-Row Tapered Roller Bearings

**Figure 1:**
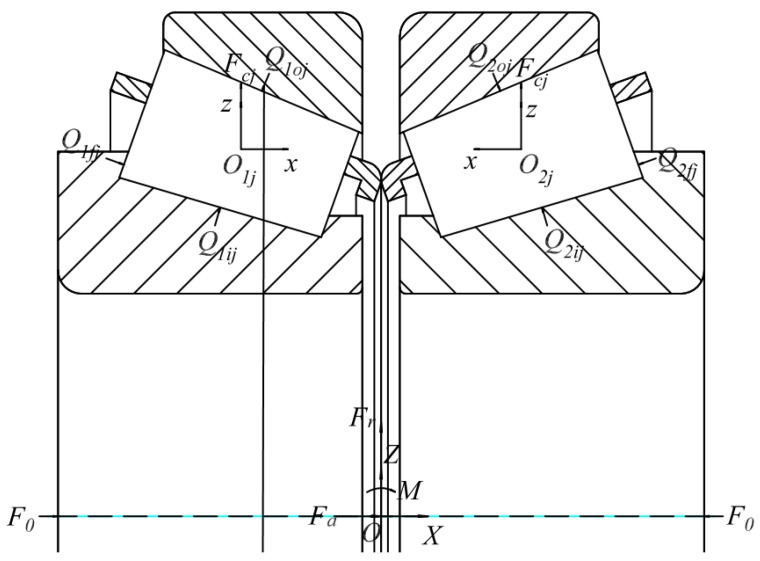
Force diagram of double-row tapered roller bearing.

Assume that the bearing as a whole is subjected to a radial outward load *F_r_*, axial outward load *F_a_*, axial preload *F*_0_, and the external bending moment load M, taking into account the centrifugal force on the roller *F_cj_*; the function is shown in Figure 1.

**Figure 2:**
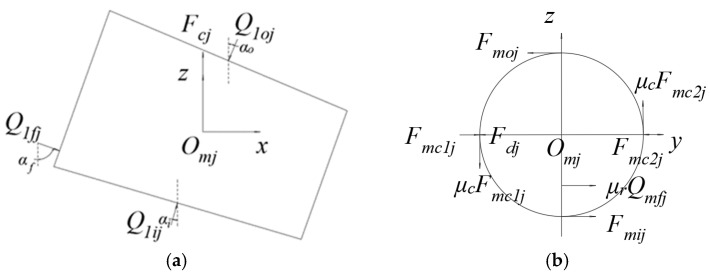
Single roller force diagram. (**a**) Roller cone surface; (**b**) Roller round surface.

For a single tapered roller with asymmetric left and right sides, the stress relationship is shown in Figure 2. *Q_moj_*_,_ *Q_mij_* and *Q_mfj_* are the roller outer raceway contact load, roller inner raceway contact load, and roller big end rib contact load, respectively; *F_mc1j_* and *F_mc2j_* is the normal force between the roller and the cage pocket; *μ_c_* and *μ_f_* is the coefficient of friction between the roller pocket and the roller rib; and *F_dj_* is the fluid resistance.

In the roller local coordinate system *o_mj_*-*xyz*, the *jth* roller in column *m* is located at *xo_mj_z*; the following equation is established for the plane force balance:

(1) $$Q_{moj}\sin\alpha_{o}-Q_{mij}\sin\alpha_{i}-Q_{mfj}\sin\alpha_{f}=0$$

(2) $$Q_{moj}\cos\alpha_{o}-Q_{mij}\cos\alpha_{i}+Q_{mfj}\cos\alpha_{f}-F_{cj}-\mu_{c}F_{mc1j}+\mu_{c}F_{mc2j}=0$$

The contact load of *F_mc1j_* and *F_mc2j_* relative to the roller and inner and outer rings is very small, so the tangential friction term between the roller and the pocket in Equation (2) can be ignored in the calculation. Therefore, the external raceway contact force *Q_moj_* for the reference and contact load *Q_mij_* and *Q_mfj_*, respectively, can be expressed as

(3) $$Q_{mij}=Q_{moj}\frac{\sin\left(\alpha_{o}+\alpha_{f}\right)}{\sin\left(\alpha_{i}+\alpha_{f}\right)}-F_{cj}\frac{\sin\alpha_{f}}{\sin\left(\alpha_{i}+\alpha_{f}\right)}$$

(4) $$Q_{mfj}=Q_{moj}\frac{\sin\left(\alpha_{o}-\alpha_{i}\right)}{\sin\left(\alpha_{i}+\alpha_{f}\right)}+F_{cj}\frac{\sin\alpha_{i}}{\sin\left(\alpha_{i}+\alpha_{f}\right)}$$

The revolution speed is *ω_c_* and its centrifugal force *F_cj_* is

(5) $$F_{cj}=\frac{1}{2}m_{s}d_{m}\omega_{c}^{2}$$

where *d_m_* is the pitch diameter; *m_s_* is the mass of a single roller.

*Q_moj_* the components in the radial and axial directions are

(6) $$Q_{mrj}=Q_{moj}\cos\alpha_{o}\cos\varphi_{j}$$

(7) $$Q_{maj}=\epsilon Q_{moj}\sin\alpha_{o}$$

where *m* is the column in which the roller is located; When *m* = 1, *ϵ* = 1; When *m* = 2, *ϵ* = 2.

Under the action of external loads, the contact load of the roller will generate a resistance moment to the bearing center *M_mj_* and the resultant moment can be expressed as

(8) $$M_{mj}=\epsilon \left(\frac{1}{2}d_{c}Q_{moj}\cos\alpha_{o}\cos\varphi_{j}+\frac{1}{2}d_{m}Q_{moj}\sin\alpha_{o}\cos\varphi_{j}\right)$$

For the bearing as a whole, assume that each row has *Z* rollers, superpose the corresponding loads of the two rows of rollers, and consider the direction of action of forces and moments to obtain the balance equation for the entire double row tapered roller bearing:

(9) $$\{\begin{array}{c} F_{r}-\sum_{m=1}^{2}\sum_{j}^{Z}Q_{moj}\cos\alpha_{o}\cos\varphi_{j}=0\\ F_{a}-\sum_{m=1}^{2}\sum_{j}^{Z}\epsilon Q_{moj}\sin\alpha_{o}=0\\ M-\sum_{m=1}^{2}\sum_{j}^{Z}\epsilon \left(\frac{1}{2}d_{c}Q_{moj}\cos\alpha_{o}\cos\varphi_{j}+\frac{1}{2}d_{m}Q_{moj}\sin\alpha_{o}\cos\varphi_{j}\right)=0 \end{array}$$

### 2.2. Relationship between Load and Deformation

**Figure 3:**
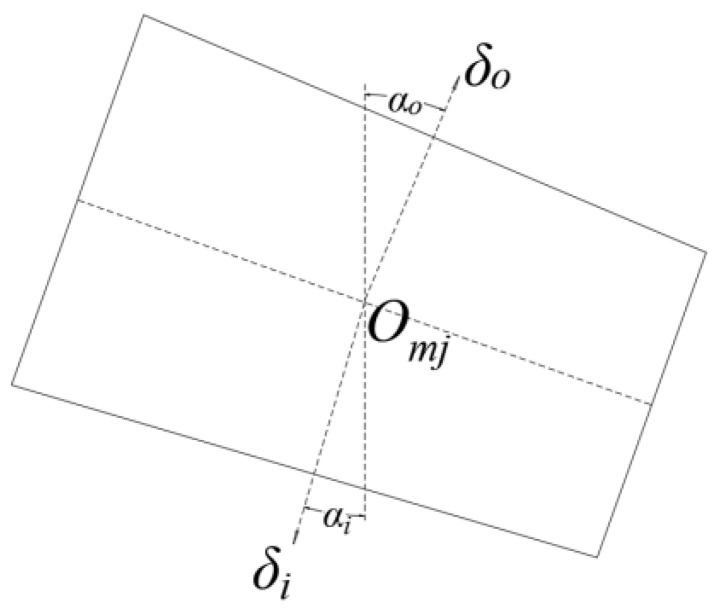
Displacement–projection relationship.

Assume that the contact displacements of the roller inner raceway and the roller outer raceway are, respectively, *δ_i_* and *δ_o_* and that the contact angles of the inner and outer raceways of tapered roller bearings are different. When calculating the total contact displacement, the displacement at the contact point of the roller outer raceway can be used as a reference to project the contact displacement of the roller inner raceway towards the normal direction of the roller outer raceway contact, as shown in Figure 3.

The contact displacement between the roller and the inner raceway according to the geometric relationship *δ_i_* and contact displacement at roller outer raceway *δ_o_* is

(10) $$\delta_{oi}=\delta_{i}\cos\left(\alpha_{o}-\alpha_{i}\right)$$

Therefore, *δ_o_* the total contact displacement in the direction is

(11) $$\delta_{n}=\delta_{o}+\delta_{i}\cos\left(\alpha_{o}-\alpha_{i}\right)$$

According to Hertz line contact, the relationship between the total contact displacement and the external raceway contact load is

(12) $$\delta_{n}=\left[G_{o}+G_{i}c_{i}^{0.9}\cos\left(\alpha_{o}-\alpha_{i}\right)\right]Q_{o}^{0.9}$$

where $c_{i}=\frac{\sin\left(\alpha_{o}+\alpha_{f}\right)}{\sin\left(\alpha_{i}+\alpha_{f}\right)}$. *G_i_* and *G_o_* are the flexibility coefficient at the contact between the inner and outer raceways, which can be obtained according to Palmgren’s correction Equation (32)

(13) $$G_{i}=4.80{\left(\frac{1-\mu_{1}^{2}}{\pi E_{1}}+\frac{1-\mu_{2}^{2}}{\pi E_{2}}\right)}^{0.9}\frac{{\left(1+k_{i}\right)}^{0.1}}{L_{e}^{0.74}D_{w}^{0.1}}$$

(14) $$G_{o}=4.80{\left(\frac{1-\mu_{1}^{2}}{\pi E_{1}}+\frac{1-\mu_{2}^{2}}{\pi E_{2}}\right)}^{0.9}\frac{{\left(1+k_{o}\right)}^{0.1}}{L_{e}^{0.74}D_{w}^{0.1}}$$

where *E* is the elastic modulus of the material; *μ* is the material Poisson’s ratio; Subscripts 1 and 2 refer to contact bodies 1 and 2 that are in contact with each other, respectively; *k_i(o)_* is the average diameter of the roller (*D_w_*) to the diameter of the inner and outer raceways (*D_ri(o)_*) ratio; *D_w_* is the averrage diameter of the roller; and *L_e_* is the effective length of the roller.

According to the relationship between Equations (12)–(14), it can be obtained that

(15) $$Q_{o}=K_{n}\delta_{n}^{1.11}$$

where $K_{n}$ is the total contact coefficient

(16) $$K_{n}={\left[G_{o}+G_{i}c_{i}^{0.9}\cos\left(\alpha_{o}-\alpha_{i}\right)\right]}^{-1.11}$$

Generally, the inner ring is assembled with the shaft by interference and is linked with the shaft, so the two inner rings of the bearing can be considered as a whole during calculation. Assume that the displacements of the inner ring relative to the outer ring under external load are, respectively, radial displacement *δ_r_*, axial displacement *δ_a_*, and angular displacement *θ*.

Radial displacement of inner ring *δ_r_* at the position angle *φ_j_* with the *j* radial displacement component of the roller is

(17) $$\delta_{mrj}=\delta_{r}\cos\varphi_{j}$$

Axial displacement of the inner ring *δ_a_* at the position angle *φ_j_* with the *j* axial displacement component of the roller is

(18) $$\delta_{maj}=\epsilon \delta_{a}$$

The inner circle produces corner *θ* at the position angle *φ_j_* with the angular displacement of the roller and is expressed as

(19) $$\theta_{mj}=\epsilon \theta \cos\varphi_{j}$$

**Figure 4:**
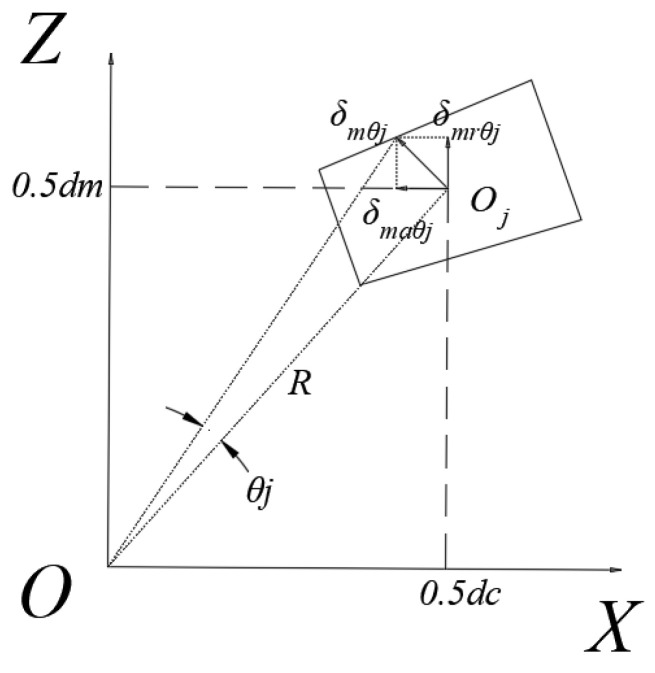
Displacement component of rotation angle *θ*.

Let the distance between the centers of the two rows of rollers be *d_c_*, then angular displacement *θ* of the resulting radial and axial displacement components (as shown in Figure 4) is

(20) $$\delta_{mr\theta j}=\frac{1}{2}d_{c}\theta_{mj}$$

(21) $$\delta_{ma\theta j}=\frac{1}{2}d_{m}\theta_{mj}$$

It is assumed that the bearing is only subjected to *F_0_* according to the equilibrium condition of axial direction force, and the relationship between contact load and displacement of combination Equation (15) is as follows

(22) $$F_{0}-ZK_{n}{\left(\delta_{0}\sin\alpha_{o}\right)}^{1.11}\sin\alpha_{o}=0$$

Obtain the displacement only when the preload is applied (*δ*_0_)

(23) $$\delta_{0}={\left(\frac{F_{0}}{ZK_{n}{\left(\sin\alpha_{o}\right)}^{2.11}}\right)}^{0.9}$$

In summary, the total normal contact deformation at the roller raceway contact of a double-row tapered roller bearing is

(24) $$\delta_{mnj}=\left(\delta_{mrj}+\delta_{mr\theta j}\right)\cos\alpha_{o}+\left(\delta_{maj}+\delta_{ma\theta j}+\delta_{o}\right)\sin\alpha_{o}$$

For the contact stiffness Equation, only the contact load is unknown, and it is necessary to obtain it. Next, calculate the contact load *Q_i(o)j_*.

### 2.3. Contact Load Calculation Process

According to the relationship between three loads and deformation, Equation (9) is a nonlinear equation set with *δ_r_*, *δ_a_* and *θ* as unknowns. The solution of the nonlinear equation set can be obtained using the iterative method, and then the contact load and contact deformation at the inner and outer raceway and the baffle can be finally obtained by substituting the solution into the corresponding equation.

During the iterative calculation process, it should be noted that due to the effect of centrifugal force, some rollers may not come into contact with the inner race raceway, while there is always a minimum normal contact load between the rollers and the outer race raceway, which can be obtained from Equation (3)

(25) $$Q_{mocj}=F_{cj}\frac{\sin\alpha_{f}}{\sin\left(\alpha_{i}+\alpha_{f}\right)}\;$$

According to the load-deformation relationship, the deformation amount generated by the minimum normal contact load is *δ_F__c_*

(26) $$\delta_{Fc}={\left(\frac{F_{cj}\sin\alpha_{f}}{K_{n}\sin\left(\alpha_{i}+\alpha_{f}\right)}\right)}^{0.9}$$

**Figure 5:**
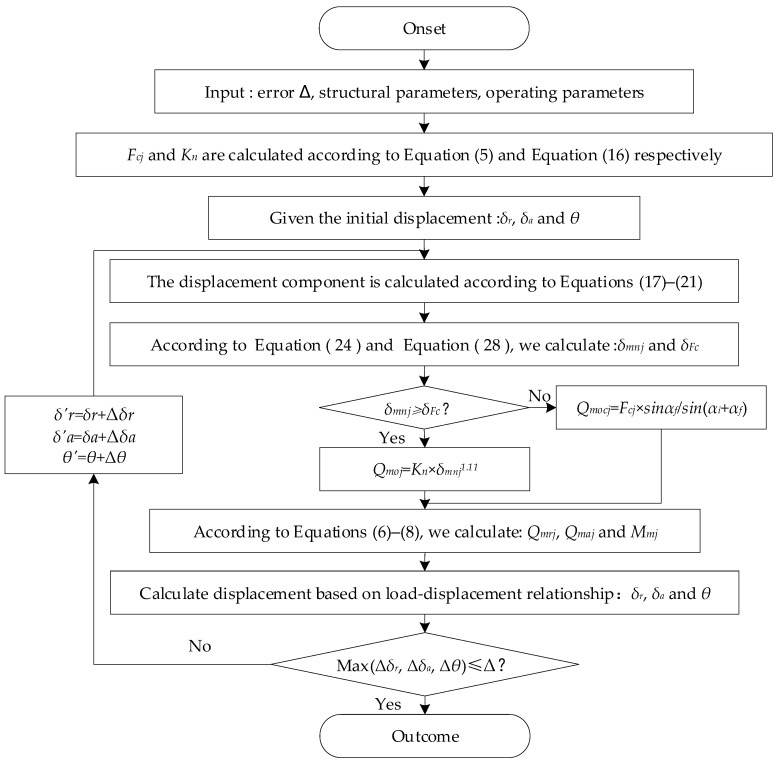
Internal contact load calculation process.

During the iteration process, taking the total contact deformation *δ_mnj_* and minimum contact deformation *δ_F__c_*, the larger value of the two is used as the new total contact deformation amount. Set the iteration error Δ = 1 × 10^−9^ and calculate the deviation between the two iteration results before and after each iteration for Δ*δ_r_*, Δ*δ_a_* and Δ*θ* until the maximum deviation is less than or equal to the set iteration error Δ, the iteration is completed and the result is given as an output. The specific calculation process is shown in Figure 5. 

### 2.4. Calculation Method of Contact Stiffness

For the contact between tapered rollers and inner and outer raceways, Luo [32] gave a load-deformation calculation equation that takes into account the diameter of the roller and raceway and the concavity and convexity of the curvature at the contact point

(27) $$\delta_{i\left(o\right)j}=1.712{\left(\frac{1-\mu_{1}^{2}}{E_{1}}+\frac{1-\mu_{2}^{2}}{E_{2}}\right)}^{0.9}\frac{{\left(1+k_{i\left(o\right)}\right)}^{0.1}}{L_{e}^{0.74}D_{w}^{0.1}}Q_{i\left(o\right)j}^{0.9}$$

The meaning of each parameter in the Equation is the same as that in Equations (13) and (14). *Q_i_*_(o)*j*_ is the contact load of the inner and outer raceways.

According to the definition of contact stiffness, the normal contact stiffness between the roller and the inner and outer raceways is obtained by deriving Equation (27) as follows:

(28) $$K_{ci\left(o\right)j}=\frac{dQ_{i\left(o\right)j}}{d\delta_{i\left(o\right)}}=\frac{L_{e}^{0.74}D_{w}^{0.1}Q_{i\left(o\right)j}^{0.1}}{1.541{\left(\frac{1-\mu_{1}^{2}}{E_{1}}+\frac{1-\mu_{2}^{2}}{E_{2}}\right)}^{0.9}{\left(1+k_{i\left(o\right)}\right)}^{0.1}}$$

The normal stiffness of tapered roller *j* can be decomposed into stiffness components along the radial direction (r) *K_rj_* and the stiffness component along the axial direction (z) *K_zj_*.

(29) $$K_{ri\left(o\right)j}=K_{ci\left(o\right)j}\cos^{2}\alpha_{i\left(o\right)}$$

(30) $$K_{zi\left(o\right)j}=K_{ci\left(o\right)j}\sin^{2}\alpha_{i\left(o\right)}$$

where *α_i(o)_* is the contact angle of the inner (outer) raceway of the bearing.

The radial and axial stiffness components of each roller and inner and outer raceways are accumulated in parallel for the entire bearing to obtain the radial stiffness of a single row of bearings *K_ra_* and axial stiffness *K_ma_.*

(31) $$K_{mr}=\sum_{j}^{Z}\frac{K_{rij}K_{roj}}{K_{rij}+K_{roj}}\cos^{2}\varphi_{j}$$

(32) $$K_{ma}=\sum_{j}^{Z}\frac{K_{rij}K_{roj}}{K_{rij}+K_{roj}}$$

where, *m* is the column of the roller, *m* = 1, 2; *φ_j_* is the position angle of the *jth* roller, *φ_j_* = 2π (*j* − 1)/Z; and *Z* is the number of rollers in each row of the bearing.

For double row bearings, the radial stiffness and axial stiffness of the first and second rows are added in parallel to obtain the radial stiffness of the double row bearings *K_r_* and axial stiffness *K_a_.*

(33) $$K_{r}=\sum_{m=1}^{2}K_{mr}$$

(34) $$K_{a}=\sum_{m=1}^{2}K_{ma}$$

## 3. Distribution Characteristics and Influence Analysis of Contact Stiffness under Complex Loading Conditions

**Figure 6:**
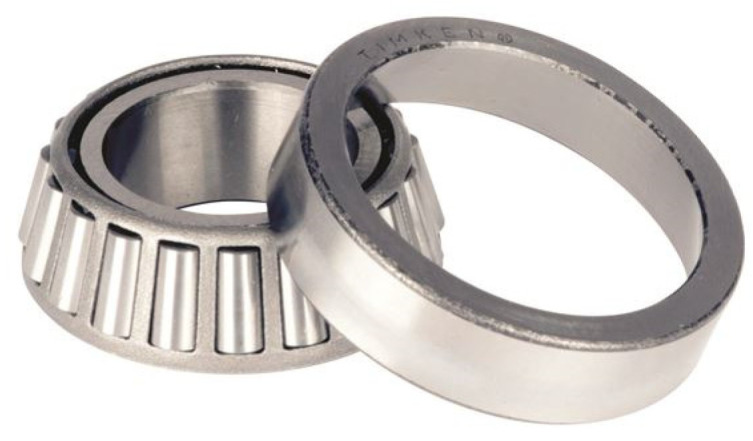
HH926700 tapered roller bearing physical drawing.

Taking the HH926700 tapered roller bearing as an example as shown in Figure 6, the distribution characteristics and influence rules of contact stiffness of tapered roller bearing under different load conditions were studied. The main structural parameters of the HH926700 bearing are shown in Table 1. The inner and outer rings are made of GCr15 bearing steel, and the material parameters are calculated using the parameter values in Table 2.

### 3.1. Effect of Radial Load on Stiffness Distribution

**Figure 7:**
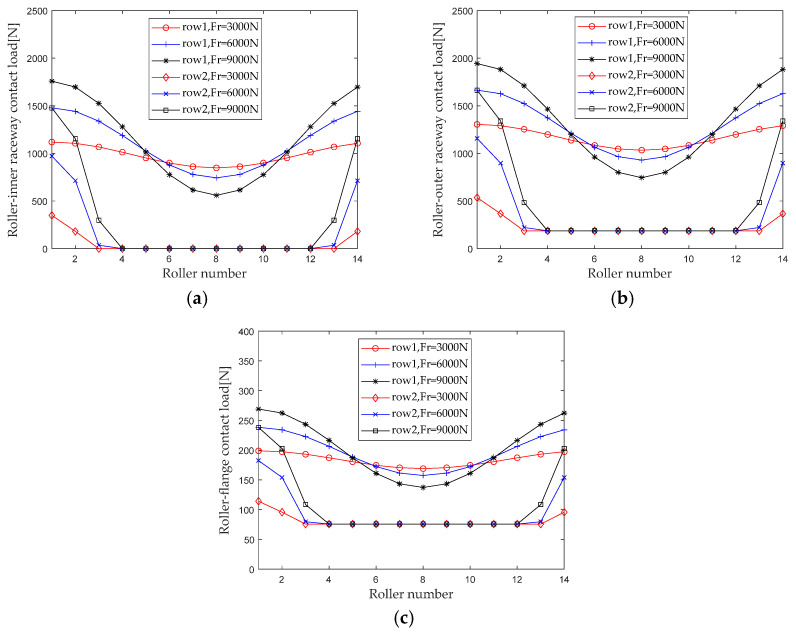
Radial load distribution. (**a**) Roller-inner raceway; (**b**) Roller-outer raceway; (**c**) Roller-flange.

When studying the influence of radial load on the internal load distribution of bearings, maintain the rotational speed, axial load, and preload, and keep the bending moment unchanged (*ω_c_* = 1200 r/min, *F_a_* = 5000 N, *F*_0_ = 100 N, *M* = 20 N × m) When the radial load *F_r_* is gradually increasing from 3000 N to 9000 N (with an interval of 3000 N), first calculate the contact loads at various locations inside the double-row tapered roller bearing under each working condition. Figure 7 shows the load distribution.

From the load distribution characteristics in Figure 6, it can be seen that under composite loads, there is a trend of increasing the number of rollers bearing radial loads. As the radial load increases, the non-uniformity of the contact load distribution between the first and second columns inside the bearing increases, and the contact load in the areas with large load becomes larger, while the contact load in the areas with small loads becomes smaller.

**Figure 8:**
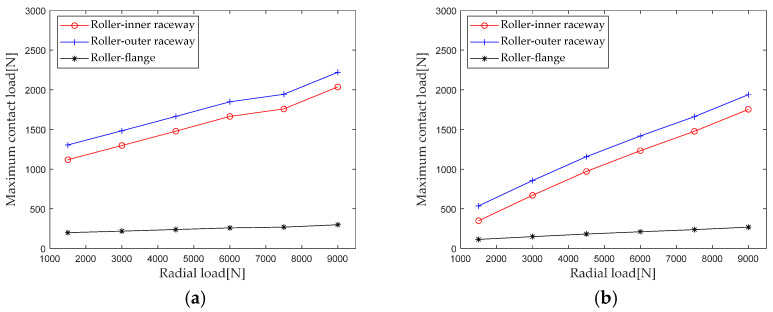
Effect of radial load on maximum contact load. (**a**) row 1; (**b**) row 2.

Generally, the maximum contact load has a significant impact on the performance such as the life of the bearing. Therefore, the maximum contact loads of the roller outer raceway, roller inner raceway, and roller rib under different radial loads are extracted for analysis, and the results are shown in Figure 8.

It can be seen that the maximum contact load at the roller outer raceway, roller inner raceway, and roller inner ring edge increases with the increase in radial load; as the radial load increases from 1500 N to 9000 N, the maximum contact loads at the first row of roller outer raceways, roller inner raceways, and roller inner ring ribs increased by 70.2% (916.2 N), 81.9% (916.2 N), and 50.6% (100.7 N), respectively. The maximum contact loads at the second row of roller outer raceways, roller inner raceways, and roller inner ring ribs increased by 261.6% (1402.5 N), 401.1% (1403.7 N), and 134.8% (154.2 N), respectively. The maximum contact load at the first row of rollers is greater than that at the second row, but the change amount and amplitude of the maximum contact load are less affected by the radial load than at the second row. This is mainly because the impact of the axial load is relatively weakened when the radial load is increased during composite loading.

**Figure 9:**
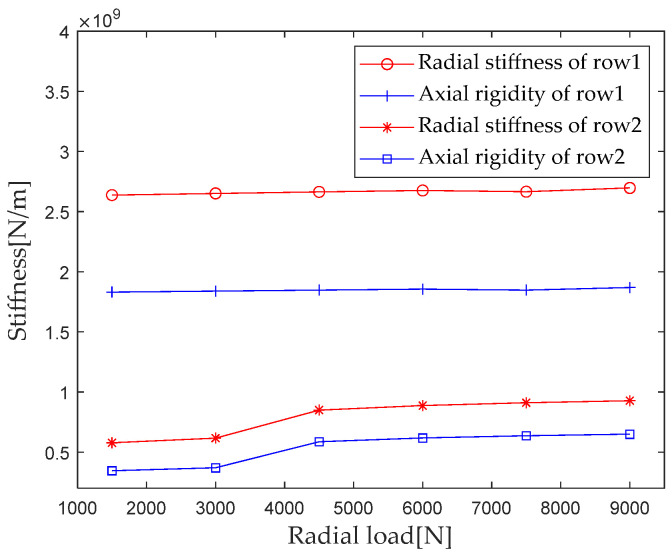
Effect of radial load on bearing stiffness component.

According to Section 2.4 Contact Stiffness, parameters such as bearing structure, material properties, and contact load are brought into the calculation equation to calculate the radial load *F_r_* when gradually increasing from 1500 N to 9000 N (with an interval of 1500 N); the first column radial stiffness, the first column axial stiffness, the second column radial stiffness, and the second column axial stiffness are analyzed, and the results are shown in Figure 9.

As can be seen from Figure 9, under the action of composite loads, the stiffness components of the first and second columns of bearings differ in magnitude, with the stiffness component of the first column being greater than the stiffness component of the second column because the load bearing capacity of the first column is greater than that of the second column under composite loads. The degree to which the stiffness components of the first and second columns are affected by radial loads is also different. The radial load increases from 1500 N to 9000 N, and the radial and axial stiffness components of the first column increase by 2.2%, while the radial and axial stiffness components of the second column increase by 60.3% and 87.6%, respectively. The stiffness components of the second column are greatly affected by radial loads, which is due to the larger change in contact load caused by the composite load on the second column compared to the first column. When the radial load increases from 3000 N to 4500 N, the contact area between the roller and raceway increases, which is the main reason for the sudden increase in the overall stiffness of the bearing mentioned above.

### 3.2. Effect of Axial Load on Stiffness Distribution

**Figure 10:**
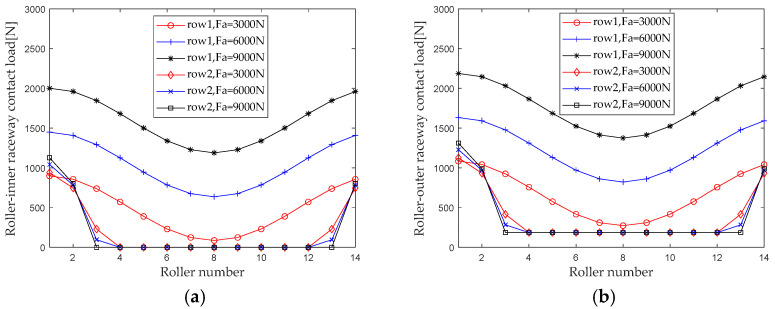

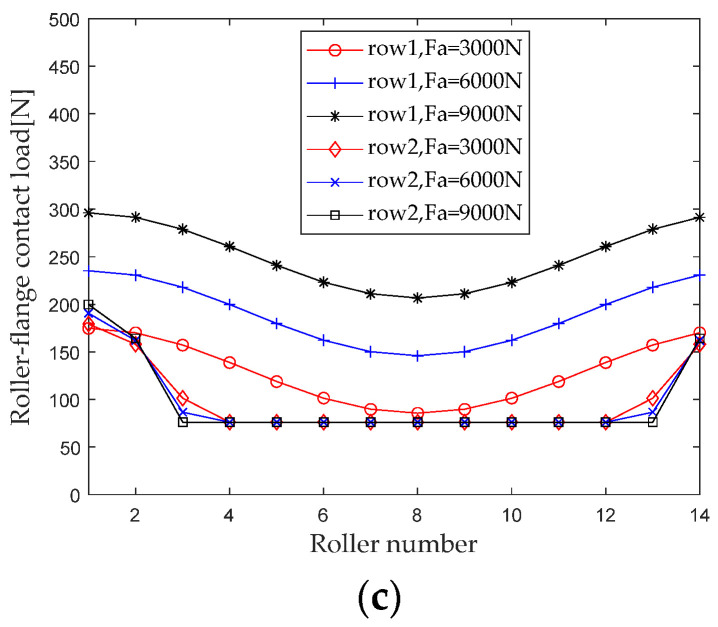
Axial load distribution. (**a**) Roller-inner raceway; (**b**) Roller-outer raceway; (**c**) Roller-flange.

When studying the effect of axial load on the internal load distribution of bearings, maintain the rotational speed, radial load, and preload, and keep the bending moment unchanged (*ω_c_* = 1200 r/min, *F_r_* = 5000 N, *F*_0_ = 100 N, *M* = 20 N×m). When the axial load *F_a_* gradually increases from 3000 N to 9000 N (with an interval of 3000 N), first calculate the contact loads at various locations inside the double-row tapered roller bearing under each working condition. Figure 10 shows the load distribution.

As can be seen from Figure 10, the difference between the first row of roller contact loads (the difference between the maximum contact load and the minimum contact load) of a double-row tapered roller bearing decreases as the axial load increases, while the difference between the second row of roller contact loads increases. The range of the bearing area at the second column shows a decreasing trend, and the contact load increases in the part of the bearing area close to the maximum contact load, while it decreases in the part of the bearing area away from the maximum contact load.

**Figure 11:**
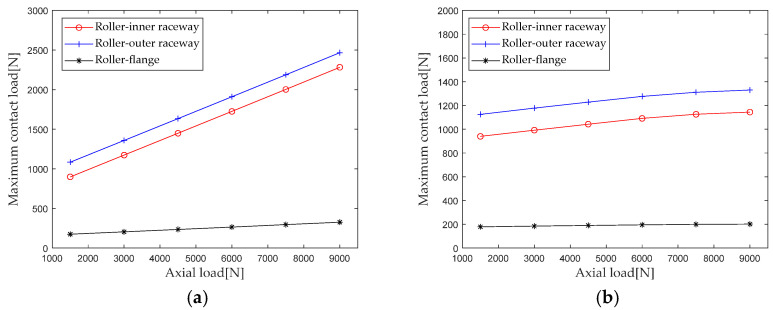
Effect of axial load on maximum contact load. (**a**) row 1; (**b**) row 2.

Extract the maximum contact loads at the roller outer raceway, roller inner raceway, and roller rib under different axial loads for analysis, and the results are shown in Figure 11. From the results in the figure, it can be seen that the maximum contact load of the first and second row rollers of the double-row tapered roller bearing increases with the increase in the axial load. As the axial load increases, the maximum contact loads of the first row of rollers at the roller outer raceway, roller inner raceway, and roller retaining edge are greater in magnitude than those of the second row of rollers. The axial load increases from 1500 N to 9000 N. The maximum contact loads of the first row of rollers at the roller outer raceway, roller inner raceway, and roller big end inner ring flange increased by 127.4% (1381.8 N), 153.8% (1382.9), and 86.9% (151.9 N), respectively. The maximum contact loads at the roller inner raceway and the roller inner ring rim increased by 18.1% (203.9 N), 21.7% (204.1 N), and 12.5% (22.4 N), respectively.

**Figure 12:**
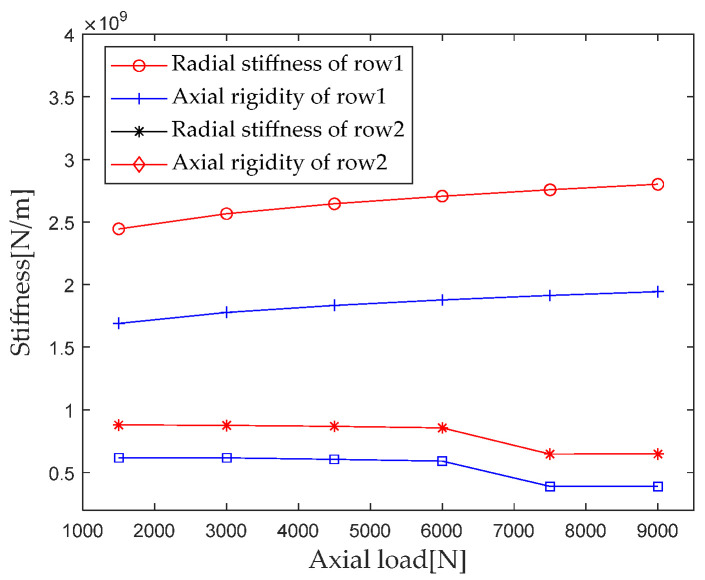
Effect of axial load on bearing stiffness component.

According to Section 2.4 Contact Stiffness, parameters such as bearing structure, material properties, and contact load are brought into the calculation equation to calculate the axial load *F_a_* when it is gradually increasing from 1500 N to 9000 N (with an interval of 1500 N); the first column radial stiffness, the first column axial stiffness, the second column radial stiffness, and the second column axial stiffness are analyzed, and the results are shown in Figure 12.

As can be seen from the results in Figure 12, for the first column of the main load, the radial and axial stiffness components increase with the increase in the axial load, with the axial load increasing from 1500 N to 9000 N, and the radial and axial stiffness increasing by 14.3% and 14.9%, respectively. In the second column, as the axial load increases, the radial and axial stiffness components first slowly increase, then decrease, and then slowly increase. When the axial load increases from 6000 N to 7500 N, the contact area between the roller and raceway decreases, which is the main reason for the sudden decrease in stiffness.

### 3.3. Effect of Bending Moment Load on Stiffness Distribution

**Figure 13:**
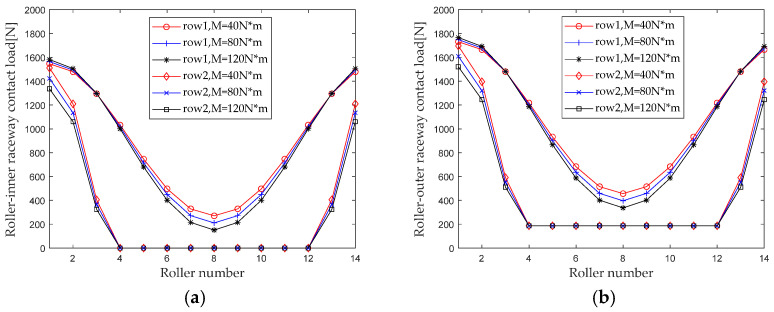

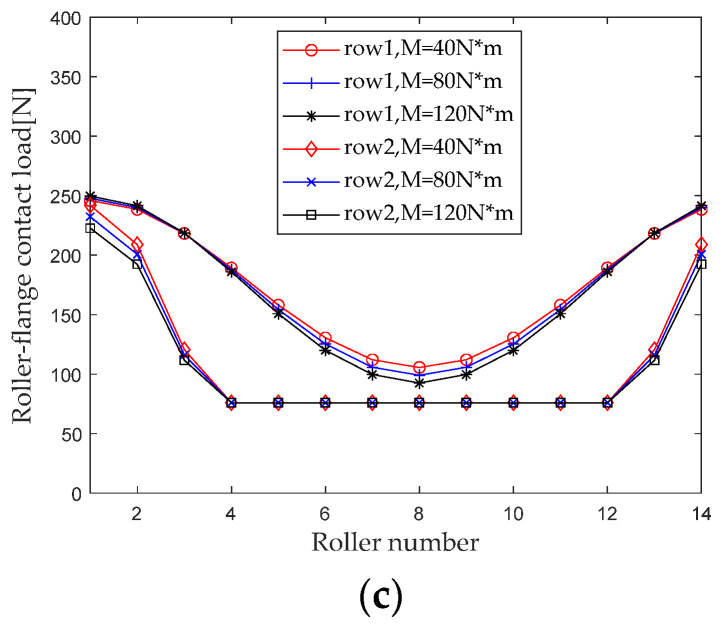
Bending moment load distribution. (**a**) Roller-inner raceway; (**b**) Roller-outer raceway; (**c**) Roller-flange.

When studying the effect of bending moment load on the internal load distribution of bearings, maintain the rotational speed, radial load, and preload, and keep the axial load unchanged (*ω_c_* = 1200 r/min, *F_r_* = 8000 N, *F*_0_ = 100 N, *F_a_* = 3000 N); the bending moment *M* gradually increased from 40 N×m to 120 N×m (with an interval of 40 N×m). First calculate the contact loads at various locations inside the double-row tapered roller bearing under each group of operating conditions. Figure 13 shows the load distribution.

From the results in Figure 13, it can be seen that the influence of the bending moment load on the load distribution at the first and second rows of rollers of a double-row tapered roller bearing has a reverse trend. The non-uniformity of the contact load distribution at the first row of rollers increases with the increase of the bending moment, while the non-uniformity of the contact load distribution at the second row of rollers decreases with the increase in the bending moment.

**Figure 14:**
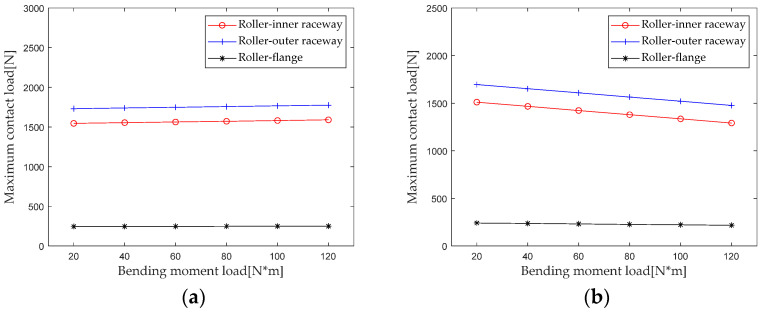
Effect of bending moment load on maximum contact load. (**a**) row 1; (**b**) row 2.

The maximum contact loads of the roller-outer raceway, roller-inner raceway and roller-rib under different axial loads are extracted for analysis. The results are shown in Figure 14. It can be seen that the influence of the bending moment on the maximum contact load of the first and second rows of rollers has an opposite trend. The maximum contact load at the roller outer raceway, roller inner raceway, and roller inner ring edge of the first row of rollers increases with the increase in the bending moment, while the maximum contact load of the second row decreases with the increase in the bending moment. When the bending moment increases from 20 N*m to 120 N*m, the maximum contact load at the first row of the roller inner raceway, roller outer raceway, and roller rib increases by 2.6% (44.7 N), 2.9% (44.6 N), and 2.0% (4.9 N), and the maximum contact load at the second row of roller inner raceway, roller outer raceway, and roller rib decreases by 14.5% (−219.3 N), 12.9% (−219.1 N), and 9.9% (−24.1 N), respectively. The maximum contact load at the second row of rollers is more affected by the change in the bending moment than the first row of rollers.

**Figure 15:**
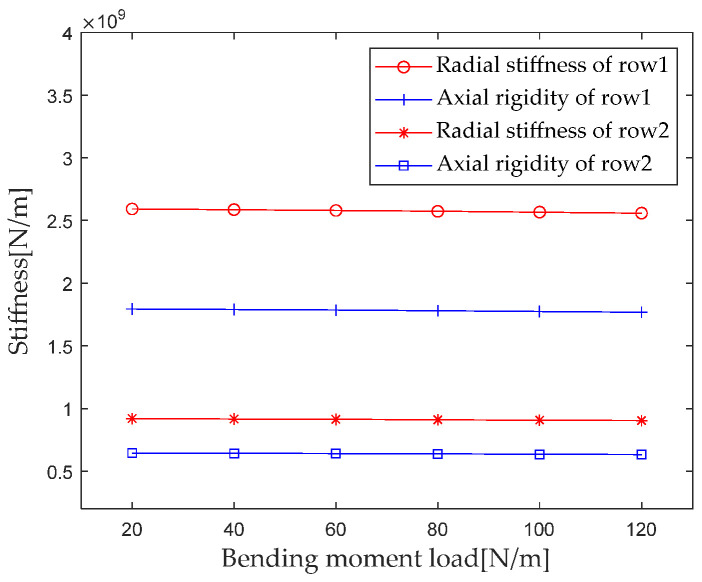
Effect of bending moment load on bearing stiffness component.

According to Section 2.4 Contact Stiffness, parameters such as the bearing structure, material properties, and contact load are brought into the calculation equation to calculate the bending moment *M* when gradually increasing it from 20 N*m to 120 N*m (with an interval of 20 N*m). The first column radial stiffness, the first column axial stiffness, the second column radial stiffness, and the second column axial stiffness are analyzed, and the results are shown in Figure 15.

From the results in Figure 15, it can be seen that the bending moment load has a small impact on the radial and axial stiffness of the first and second columns. For the first column, when the bending moment load increases from 20 N*m to 120 N*m, the radial and axial stiffness decreases by 1.2% and 1.1%, respectively. For the second column, when the bending moment load increases from 20 N*m to 120 N*m, the radial and axial stiffness decreases by 1.7% and 1.9%, respectively.

### 3.4. Effect of Rotational Speed on Stiffness Distribution

**Figure 16:**
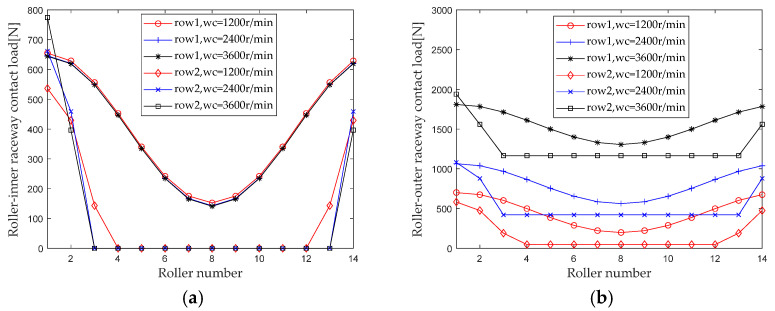

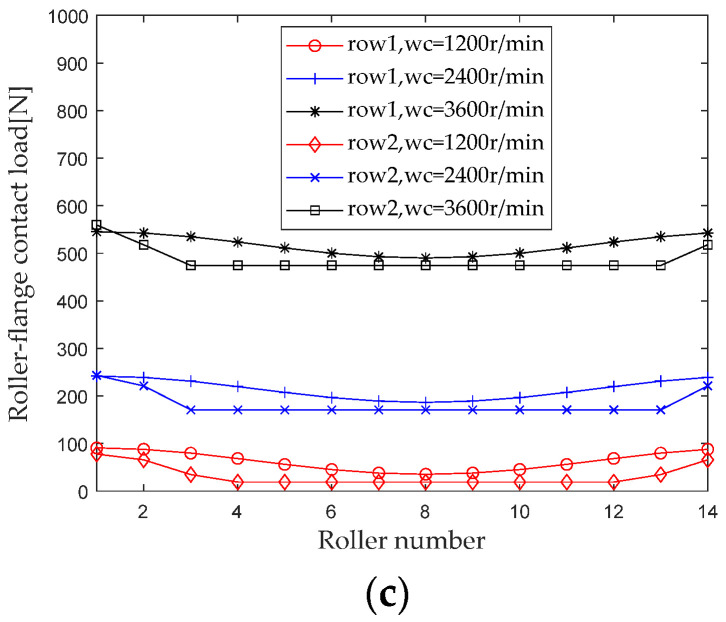
Speed load distribution. (**a**) Roller-inner raceway; (**b**) Roller-outer raceway; (**c**) Roller-flange.

When studying the effect of rotational speed on the internal load distribution of bearings, maintain the bending moment, radial load, and preload, and keep the axial load unchanged (*M* = 20 N*m, *F_r_* = 3000 N, *F_0_* = 100 N, *F_a_* = 1500 N). For the rotational speed *ω_c_* when gradually increasing from 1200 r/min to 3600 r/min (with an interval of 1200 r/min), first calculate the contact loads at various locations inside the double-row tapered roller bearing under each group of operating conditions. Figure 16 shows the load distribution.

As can be seen from Figure 16, when the rotational speed increases, the contact loads of the roller outer raceway and the roller rib at each roller show an increasing trend due to the centrifugal force, while the contact loads of the roller inner raceway slightly decrease.

**Figure 17:**
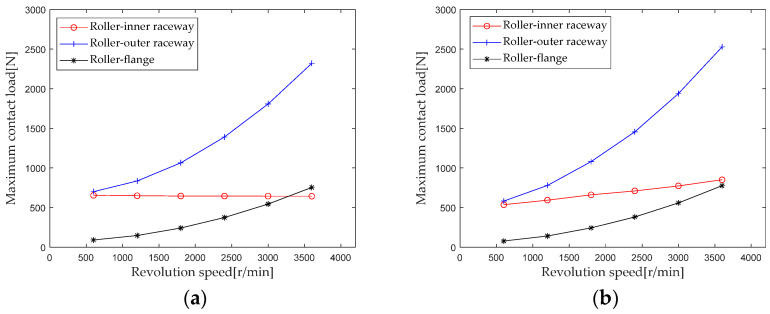
Effect of speed load on maximum contact load. (**a**) row 1; (**b**) row 2.

Extract the maximum contact loads at the roller outer raceway, roller inner raceway, and roller rib under different axial loads for analysis, and the results are shown in Figure 17. Comparing the results of the first and second columns, it can be seen that the influence trend and intensity of rotational speed on the maximum contact load at the same position of the first and second columns are basically consistent. Comparing the maximum contact loads at the roller inner raceway, roller outer raceway, and roller rib at the same column, it can be seen that considering the centrifugal force, the rotational speed has the greatest impact on the contact load of the roller outer raceway.

**Figure 18:**
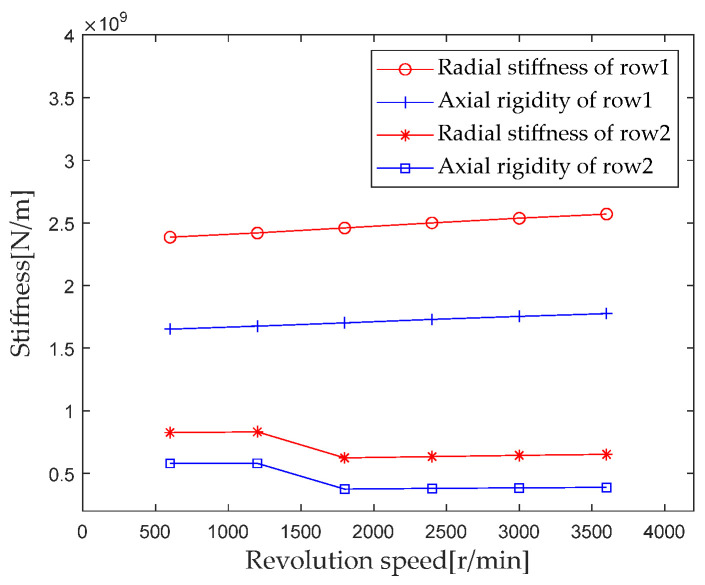
Effect of speed load on bearing stiffness component.

According to Section 2.4 Contact Stiffness, parameters such as bearing structure, material properties, and contact load are brought into the calculation equation to calculate the rotational speed *ω_c_* when gradually increasing from 1200 r/min to 3600 r/min (with an interval of 600 r/min). The first column radial stiffness, the first column axial stiffness, the second column radial stiffness, and the second column axial stiffness are analyzed, and the results are shown in Figure 18.

As can be seen from the results in Figure 18, for the first column, the radial and axial stiffness components increase as the rotational speed increases, increasing from 600 r/min to 3600 r/min, and the radial and axial stiffness increases by 7.5% and 7.9%, respectively. In the second column, the radial and axial stiffness components show a trend of first slowly increasing, then decreasing, and then slowly increasing as the rotational speed increases. When the rotational speed increases from 1200 r/min to 1800 r/min, the contact area between the roller and the raceway decreases, which is the main reason for the sudden decrease in stiffness.

### 3.5. Effect of Preload on Stiffness Distribution

**Figure 19:**
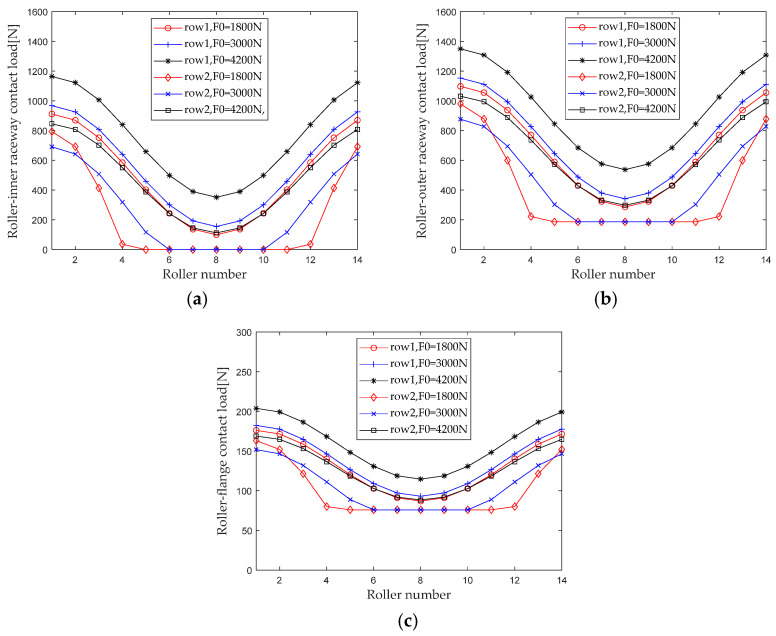
Preload load distribution. (**a**) Roller-inner raceway; (**b**) Roller-outer raceway; (**c**) Roller-flange.

When studying the influence of preload on the internal load distribution of bearings, maintain the bending moment, radial load, and rotational speed, and keep the axial load unchanged (*M* = 20 N*m, *F_r_* = 5000 N, *ω_c_* = 1200 r/min, *F_a_* = 1500 N). When the preload gradually increases from 1800 N to 4200 N (with an interval of 1200 N), first calculate the contact loads at various locations inside the double-row tapered roller bearing under each working condition. Figure 19 shows the load distribution.

When the axial preload is small, the bearing only partially bears the load of the roller under the combined load. When the preload increases to a certain value, the rollers in the first and second rows gradually change from partially loaded to fully loaded. The maximum contact load of the roller outer raceway, roller inner raceway, and roller rib increases significantly with increasing preload.

**Figure 20:**
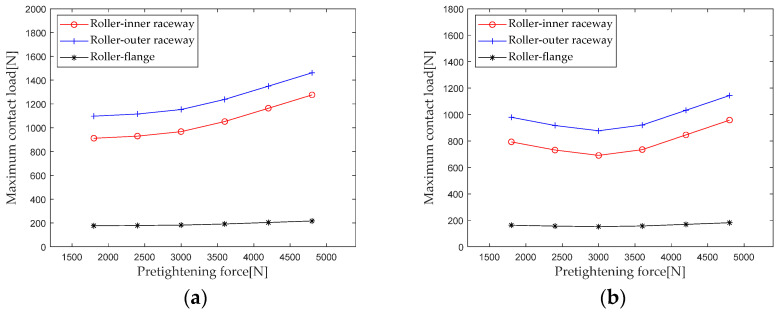
Effect of pretightening force on maximum contact load. (**a**) row 1; (**b**) row 2.

Extract the maximum contact loads at the roller outer raceway, roller inner raceway, and roller rib under different preloads for analysis, and the results are shown in Figure 20. As can be seen from Figure 20, the maximum contact load of double-row tapered roller bearings shows different trends in the first and second columns. For the first row of rollers, when the preload is not large (≤3000 N), the maximum contact load of the roller inner raceway and the roller outer raceway first slowly increases, and then as the preload further increases (>3000 N), the maximum contact load of the roller inner raceway and the roller outer raceway rapidly increases. For the second row of rollers, the maximum contact load presents a trend of decreasing first and then increasing. The maximum contact load between the second row of rollers and the inner and outer raceways presents a minimum value when the preload is about 3000 N. Therefore, there is an optimal preload that minimizes the maximum internal contact load of the bearing while other load conditions remain unchanged. This effect of preload on internal contact loads can be used as a reference for applying the best preload to bearings.

**Figure 21:**
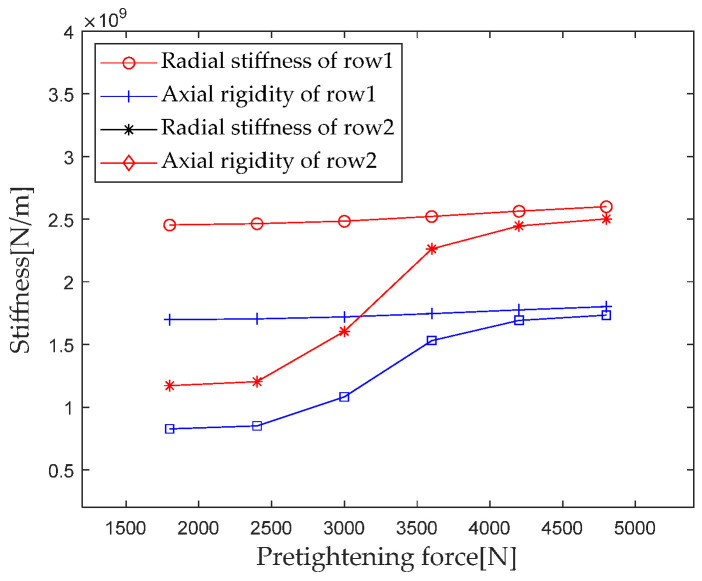
Effect of pretightening force on bearing stiffness component.

According to Section 2.4, the parameters such as bearing structure, material properties, and contact load are brought into the calculation equation. When the preload gradually increases from 1800 N to 4200 N (with an interval of 600 N), the first column radial stiffness, the first column axial stiffness, the second column radial stiffness, and the second column axial stiffness are calculated and analyzed. The results are shown in Figure 21.

From Figure 21, it can be seen that the stiffness component of the first column shows a slowly increasing trend with the increase of the preload, while the stiffness component of the second column shows a slow increase at first and then a rapid increase. The above trend of stiffness change is mainly due to the increase in preload, which gradually changes the second row of rollers from partially loaded to fully loaded, resulting in a rapid change in stiffness.

### 3.6. Effect of Deflection Angle on Stiffness Distribution

**Figure 22:**
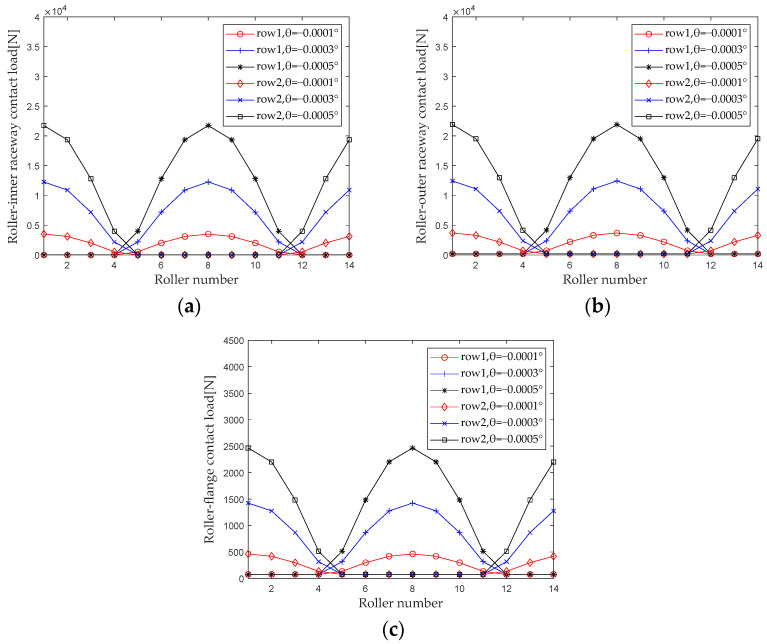
Deflection angle load distribution. (**a**) Roller-inner raceway; (**b**) Roller-outer raceway; (**c**) Roller-flange.

When studying the effect of deflection angle on the internal load distribution of bearings, the axial displacement is 0, the radial displacement is 0, and the rotational speed is constant (*δ_r_* = 0, *δ_a_* = 0, *ω_c_* = 1200 r/min, *F*_0_ = 100 N). When the deflection angle gradually increases from −0.0001° to −0.0005° (with an interval of −0.0002°), first calculate the contact loads at various locations inside the double-row tapered roller bearing under each group of operating conditions. Figure 22 shows the load distribution.

From Figure 22, it can be seen that the influence of deflection angle on the load distribution of the first row of rollers and the second row of rollers is completely different. When the first row of rollers is loaded, the rollers with corresponding numbers in the second row are not loaded, and when the second row of rollers is loaded, the rollers with corresponding numbers in the first row are not loaded. The maximum contact load of the roller outer raceway, roller inner raceway, and roller rib increases significantly with the increase in the deflection angle.

**Figure 23:**
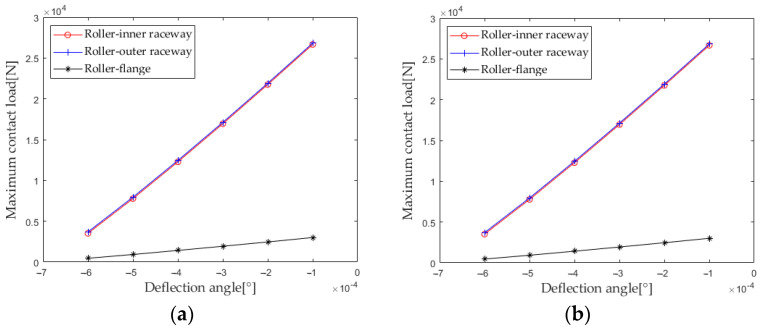
Effect of deflection angle on maximum contact load. (**a**) row 1; (**b**) row 2.

Extract the maximum contact loads at the lower roller outer raceway, roller inner raceway, and roller rib under different deflection angles for analysis, and the results are shown in Figure 23. As can be seen from Figure 23, the maximum contact loads of each contact zone in the first and second columns are completely consistent, and the maximum contact loads of the roller inner raceway and the roller outer raceway are almost equal. As the deflection angle increases, the maximum contact loads of each contact zone increase, and the increasing trend of the maximum contact loads of the roller inner raceway and the roller outer raceway is significantly greater than the maximum contact loads at the roller rib.

**Figure 24:**
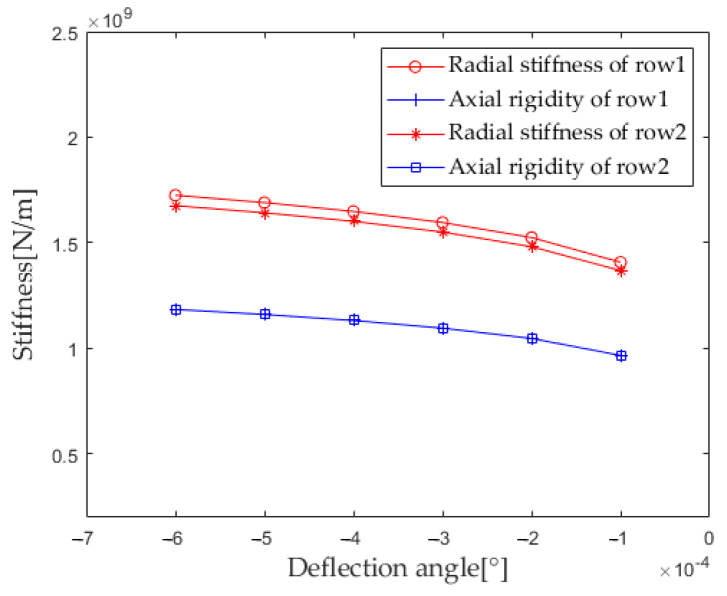
Effect of deflection angle on bearing stiffness component.

According to Section 2.4 Contact Stiffness, the parameters such as bearing structure, material properties, and contact load are brought into the calculation equation. When the deflection angle gradually increases from −0.0001° to −0.0006° (with an interval of −0.0001°), the first column radial stiffness, the first column axial stiffness, the second column radial stiffness, and the second column axial stiffness are calculated and analyzed. The results are shown in Figure 24.

From the results in Figure 24, it can be seen that the stiffness of the first and second columns increases with the increase of the deflection angle and the trend is almost consistent. The axial stiffness of the first and second columns is completely equal, and the radial stiffness of the first column is slightly greater than the radial stiffness of the second column.

## 4. Simulation Verification and Comparison

**Figure 25:**
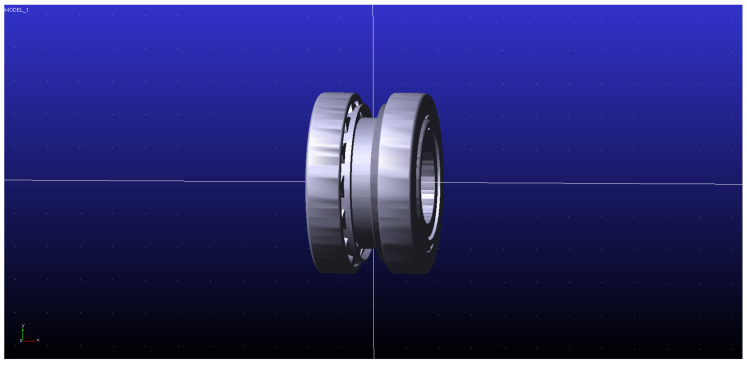
Adams simulation model diagram.

Simulation is implemented on Adams2020. The double-row tapered roller bearing model is established in Adams2020 software, as shown in Figure 25. Structural parameters and material property parameters are the same as shown in Table 1 and Table 2.

**Figure 26:**
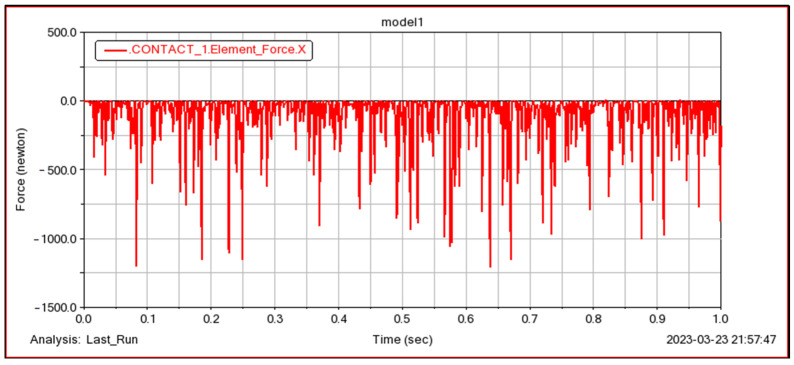
Adams solution results under the first condition.

In order to verify the accuracy of the model proposed in this paper, in the Adams simulation, the bearing is carried out under the following three different working conditions. The solution results under the first condition are shown in Figure 26 (because the length is too long, this paper does not list all the solution results). The internal contact load is further extracted and compared with the model proposed in this paper, as shown in Table 3.

From the comparison of the results in Table 3, it can be seen that the calculation results of the contact load at the roller-inner raceway, roller-outer raceway and roller-inner rib are very close to the Adams simulation results: under the three working conditions shown, the maximum error of the internal contact load is within 8%, which verifies the accuracy of the model in this paper. In addition, the calculation results of this model are slightly larger than the Adams simulation results, mainly because the high speed is considered in the model.

## 5. Conclusions and Prospect

Aiming at the problem of contact stiffness of double-row tapered roller bearings, an equation for the contact stiffness of double-row tapered roller bearings is derived based on the calculation of load distribution. The influence of complex loads on the contact stiffness of bearings is studied through numerical calculations, and the following conclusions are obtained:

(1) The contact load calculation results of roller-inner raceway, roller-outer raceway and roller-rib obtained using the model and method proposed in this paper are in good agreement with the Adams simulation results, and the maximum relative error is within 8%, which can provide some guidance for engineering application.
(2) Under a radial load, as the radial load increases, when the contact between the roller and the raceway increases, the second row of bearings will have a “sudden increase phenomenon”. Under an axial load, as the radial load increases and the contact between the roller and raceway decreases, the second row of bearings will experience a “sudden decrease phenomenon”. The influence of bending moment load on stiffness is very small. When the rotational speed increases, the contact between the roller and the raceway will decrease, and the second row of bearings will have a “sudden decrease phenomenon”. As the axial preload increases, the second row of rollers gradually changes from partially loaded to fully loaded, and the stiffness increases rapidly. The axial preload has the greatest impact on the bearing stiffness. As the deflection angle increases, the stiffness steadily increases, and the axial stiffness of the first and second columns is completely equal.
(3) There is an optimal preload that minimizes the maximum contact load of the roller when other load conditions remain unchanged for a double-row tapered roller bearing. This effect of preload on the maximum contact load can provide guidance for applying preload in engineering applications.

Future work plan: The authors will build a double-row tapered roller bearing test platform to further verify the validity and accuracy of the method and model. Based on the work of this paper, the influence of the assembly process of bearing and rotor on the dynamic stiffness of double-row tapered roller bearing is studied.

## Figures and Tables

**Table 1:** HH926700 bearing structure parameters.

Parameter	Unit	Numerical Value
Small end diameter of roller *D_w1_*	mm	30.27
Large end diameter of roller *D_w2_*	mm	36.74
Large end spherical radius of roller *R_s_*	mm	109.05
Effective roller length *L_e_*	mm	57.02
Bearing inner ring diameter *d*	mm	120.65
Bearing outer ring diameter *D*	mm	273.05
Diameter of inner raceways *D_i_*	mm	167.35
Diameter of outer raceways *D_o_*	mm	230.51
Roller-Outer raceway contact angle *α_o_*	deg	22.54
Roller-Inner raceway contact angle *α_i_*	deg	16.24
Contact angle of roller big end-flange *α_f_*	deg	70.20
The distance between two roller centers *d_c_*	mm	75.96
Number of rollers Z	ind	14
Roller quality *m_s_*	kg	0.126
Pitch diameter *d_m_*	mm	198.93

**Table 2:** GCr15 material parameters.

Parameter	Unit	Numerical Value
Density *ρ*	kg/m^3^	7810
Young’s modulus *E*	Mpa	2.1 × 10^5^
Coefficient of linear expansion *α_r_*	/°C	12.03 × 10^6^
Poisson ratio *μ*	-	0.3

**Table 3:** Comparison of this model and Adams simulation results.

Work Condition		Maximum Contact Load of Outer Raceway(N)		Maximum Contact Load of Inner Raceway(N)		Maximum Contact Load of Flange(n)		Maximal Relation Error
		Model in This Paper	Adams Simulation	Model in This Paper	Adams Simulation	Model in This Paper	Adams Simulation	
*ω_c_* = 1200 r/min	*F_r_* = 1500 N, *F_a_* = 5000 N, *F*_0_ = 100 N, *M* = 20 N*m	1304.8	1207.8	1119.3	1039.9	198.9	185.0	7.4%
	F_r_ = 5000 N, *F_a_* = 1500 N, *F*_0_ = 100 N, *M* = 20 N*m	1084.6	1002.2	898.9	836.0	174.7	162.8	7.6%
	F_r_ = 8000 N, *F_a_* = 3000 N, *F*_0_ = 100 N, *M* = 20 N*m	1731.3	1624.0	1546.1	1462.6	245.8	237.9	6.2%

## Data Availability

Not applicable.
